# Effect of Venom from the Jellyfish *Nemopilema nomurai* on the Silkworm *Bombyx mori* L

**DOI:** 10.3390/toxins7103876

**Published:** 2015-09-24

**Authors:** Huahua Yu, Rongfeng Li, Xiaolin Chen, Yang Yue, Ronge Xing, Song Liu, Pengcheng Li

**Affiliations:** Institute of Oceanology, Chinese Academy of Sciences, 7 Nanhai Road, Qingdao 266071, China; E-Mails: yuhuahua@qdio.ac.cn (H.Y.); rongfengli@qdio.ac.cn (R.L.); chenxl@qdio.ac.cn (X.C.); buqun08@163.com (Y.Y.); xingronge@qdio.ac.cn (R.X.); sliu@qdio.ac.cn (S.L.)

**Keywords:** venom, jellyfish, *Nemopilema nomurai*, toxicity, *Bombyx mori* L.

## Abstract

The silkworm *Bombyx mori* L. (*B. mori*) has a significant impact on the economy by producing more than 80% of the globally produced raw silk. The exposure of silkworm to pesticides may cause adverse effects on *B. mori*, such as a reduction in the production and quality of silk. This study aims to assay the effect of venom from the jellyfish *Nemopilema nomurai* on growth, cuticle and acetylcholinesterase (AChE) activity of the silkworm *B. mori* by the leaf dipping method. The experimental results revealed that the four samples caused neither antifeeding nor a lethal effect on *B. mori*. The sample SFV inhibited *B. mori* growth after 6 days of treatment in a dose-dependent manner. The samples SFV, DSFV and Fr-1 inhibited the precipitation and synthesis of chitin in the cuticle after 12 and 14 days of treatment. In the case of the four samples, the AChE was significantly improved after 14 days of treatment.

## 1. Introduction

The silkworm *Bombyx mori* L. (*B. mori*) has an impact on the global economy, especially regarding foreign trade in China. *B. mori* produces more than 80% of the globally produced raw silk [1]. The silkworm growth environment is an important factor that affects the quality of silk. In recent years, accidents frequently occurred in silkworm rearing as a result of pesticide applications to field crops, possibly close to mulberry trees. Pesticide poisoning has become a direct threat to silkworm production and causes more than a 30% reduction in the annual production of raw silk in China [2]. As a result, the effect of various substances as potential pesticides on silkworm has become an interesting issue. Some studies on the effect of pesticides and plant extracts on *B. mori* have been reported, especially dealing with toxicity, retardation of development and growth, mortality and food utilization [3,4,5,6]. In addition, the silkworm is very sensitive to pesticide and can be employed as an indicator to evaluate the impact of substances as potential pesticides on environmental safety, which is part of the pesticide registration requirements in China and Japan [7,8].

*Nemopilema nomurai* Kishinouye (*N. nomurai*), 1922, is one of the most toxic types of giant jellyfish [9]. It has bloomed in the southern Yellow Sea and the northern East China Sea and its distribution is related to temperature and salinity [10,11]. Most *N. nomurai* specimens are considered to be worthless and are thrown into the sea when collected in trawls due to their high water content (approximately 95%), fragility and difficulty of processing. However, jellyfish venom has been proven to have insecticidal activity and *Stephanitis pyri* Fabricius may be a potential target pest of jellyfish venom [12], which can be reasonably considered as a potential pesticide.

Venomous animals produce diverse chemical cocktails for defense, prey capture, competitor deterrence, and/or extraoral digestion, and have been proven to be a valuable source of pharmacologically active compounds and a tool to study cell physiology [13,14]. Reports about the potential application of venom in agriculture have primarily focused on the insecticidal activity of spider and scorpion venom [15,16,17]. However, no reports have addressed the effect of jellyfish venom on silkworm.

In an attempt to develop new tools as pesticides that do not compromise silk production and quality, the present study aims to assay the effect of *N. nomurai* venom on silkworm *B. mori*, which is selected as a model, by hypothesizing that this venom may affect growth, cuticle and the acetylcholinesterase (AChE) activity of a non-target insect.

## 2. Results and Discussion

### 2.1. Effect of Jellyfish Venom on Silkworm Growth

It was found that the third instar silkworm molted to the fourth instar on the third day, and the fourth instar silkworm molted to the fifth instar on the eighth day. Table 1 reports the effect of jellyfish venom on silkworm growth. The experimental results indicated that the four samples SFV, DSFV, Fr-1 and Fr-2 (SFV: Full venom extracted by phosphate buffer solution (0.01 mol/L, pH 6); DSFV: SFV dialysed in phosphate buffer solution (0.01 mol/L, pH 6); Fr-1: Precipitate from SFV subjected to 30% (NH4)_2_SO4; Fr-2: Precipitate from supernatant subjected to 60% (NH4)_2_SO4.) had no antifeeding effect on food intake of *B. mori* and no *B. mori* died. SFV inhibited *B. mori* growth after 6 days of treatment. The higher the SFV concentration, the stronger the inhibiting effect. Conversely, SFV and DSFV facilitated the growth of *B. mori* after 2 and 4 days of treatment, respectively. Fr-1 and Fr-2 facilitated the growth of *B. mori* after 2, 4 and 6 days of treatment. After the experiment, the remnant silkworms were fed with mulberry leaves without jellyfish venom; they subsequently cocooned.

**Table 1:** Effect of jellyfish venom on silkworm growth.

Sample	Concentration (µg/mL)	IRM (%)		
		2 days	4 days	6 days
Control	0	102.16 ± 3.91	207.48 ± 6.95	620.92 ± 45.61
SFV	188	121.94 ± 20.12	234.47 ± 21.33	509.03 ± 41.18
	18.8	126.57 ± 15.23	247.73 ± 29.96	578.80 ± 90.30
	3.76	116.63 ± 12.16	259.13 ± 30.66	586.40 ± 75.50
DSFV	196	117.13 ± 7.21	241.62 ± 6.80 *	505.02 ± 38.90
	19.6	169.70 ± 18.00 *	287.93 ± 30.28 *	695.04 ± 9.63
	3.92	151.24 ± 20.07	260.57 ± 21.60	688.07 ± 77.55
Fr-1	81	161.99 ± 31.30	341.79 ± 60.01	902.58 ± 152.19
	8.1	159.54 ± 7.75 **	288.90 ± 18.99 **	691.62 ± 27.71
	1.62	159.83 ± 27.52	291.77 ± 50.62	700.05 ± 83.63
Fr-2	173	131.23 ± 5.64 *	265.48 ± 13.28 *	642.80 ± 22.32
	17.3	151.86 ± 9.92 *	271.90 ± 12.54 *	717.76 ± 38.71
	3.46	134.95 ± 13.54	271.78 ± 33.83	704.09 ± 68.69

The third instar silkworms were fed with mulberry leaves that were soaked in the SFV, DSFV, Fr-1 and Fr-2 samples for 7 days. Each sample involved three dosages and phosphate buffer solution (0.01 mol/L, pH 6) was employed as control. The experiment involved three replicates per dosage and the control. 40 third instar silkworms were utilized in each replicate. The increment rate of mass (IRM) was counted after 2, 4 and 6 days of treatment, according to the following formula: IRM% = (mt − m0) × 100/m0, mt: mass after treatment; m0: mass before treatment. * *p* < 0.05, ** *p* < 0.01, *n* = 3.

The quality of mulberry leaves is the major factor that affects the quality and quantity of cocoons produced by the silkworm [5]. Generally, the toxic stress condition of insecticides alters, the metabolic function of the silkworm to satisfy the required energy demands, which may drastically reduce the silkworm weight. However, all venom samples facilitated the growth of *B. mori*. Jellyfish venom is mainly composed of proteins [18] and may provide *B. mori* with nutrition for growth. In addition, according to our previous studies [19,20], the protein molecular weights of Fr-1 and SFV ranged from 90 kDa to 300 kDa and from 10 kDa to 300 kDa, respectively. The protein molecular weight of Fr-2 was similar to the protein molecular weight of SFV, but the color of the bands from 10 kDa to 90 kDa was lighter than the color bands of SFV. The four samples had the growth factors of *B. mori*; the growth factors were primarily evident in the proteins with a minimum molecular weight of 90 kDa. For the metal ions, such as K^+^, Na^+^, Mg^2+^ and Ca^2+^ in the undialyzed samples, SFV may affect the growth of *B. mori*. DSFV at 196 µg/mL inhibited *B. mori* growth after 6 days of treatment, which may be related to the physiology of the silkworm.

The effect of extensively applied and commercial-grade insecticides such as iminoctadine, fentrothion, ethion and destruxin A, on the silkworm, including subacute and delayed toxicity, carbohydrate and protein metabolism in hemolymph and fat body, digestive enzymes, and food utilization were investigated [4,21,22,23,24]. In addition, the effects of some extracts from plants were also assayed. In particular, myricetin, crude extract from Waxberry leaves and the plant *Datura* *suaveolens* had no toxic effect on *B. mori* but had an antifeeding effect [25,26]. Extracts from medicinal *Euphorbia fisheriana* and *Chelidoniummaius* L. had a toxic effect on *B. mori* [26]. Jatropherol I from *Jatropha curcas* seed oil had a strong stomach toxicity effect on *B. mori*, which inhibited its growth [27]. Thus, venom from jellyfish *N. nomurai* had no toxic effect on non-target insect silkworm *B. mori* and was safer than insecticides and extracts from plants.

### 2.2. Effect of Jellyfish Venom on Silkworm Cuticle

Table 2 shows the effect of jellyfish venom on silkworm cuticle. The samples SFV, DSFV and Fr-1 inhibited the precipitation and synthesis of chitin in the cuticle after 12 and 14 days of treatment. However, the inhibition was not dependent on the concentration. For the concentrations of 117 and 11.70 µg/mL, the sample SFV elicited the most significant inhibition (*p* < 0.01) after 12 days of treatment. The sample Fr-2 had no distinct effect on the chitin in the cuticle. The four samples had no regular effect on the protein and ash in the cuticle after 12 days of treatment. However, the four samples increased the content of protein and reduced the content of ash in the cuticle after 14 days of treatment. The protein content of the control after 14 days of treatment was significantly lower than the protein content of the control after 12 days of treatment, whereas the ash content of the control after 14 days of treatment was significantly higher than the control one after 12 days of treatment, indicating that the four samples inhibited the sediment of the mineral substance in the cuticle.

**Table 2:** Effect of jellyfish venom on silkworm cuticle.

Sample	Concentration (µg/mL)	12 days			14 days		
		Protein (%)	Chitin (%)	Ash (%)	Protein (%)	Chitin (%)	Ash (%)
Control	0	46.21 ± 2.54	51.93 ± 0.73	1.86 ± 2.33	23.04 ± 13.76	56.94 ± 4.05	20.02 ± 11.99
SFV	117	50.97 ± 2.36 *	46.25 ± 0.89 **	2.78 ± 1.82	34.63 ± 9.02	56.30 ± 5.36	9.07 ± 3.91
	11.7	53.58 ± 3.45	41.35 ± 0.77 **	5.07 ± 3.64	48.02 ± 11.93	50.18 ± 0.89	1.79 ± 1.19
	2.34	45.02 ± 10.11	44.41 ± 6.12	10.57 ± 4.26	36.77 ± 8.13	54.67 ± 2.02	8.55 ± 7.19
DSFV	294	51.52 ± 1.77	47.26 ± 2.43	1.22 ± 0.72	36.46 ± 7.66	53.26 ± 1.56	10.28 ± 7.14
	29.4	44.19 ± 6.79	49.66 ± 0.59	6.15 ± 6.49	47.67 ± 2.63	50.49 ± 3.30 *	2.05 ± 1.77
	5.88	50.61 ± 3.29	43.94 ± 2.24 *	5.45 ± 1.22	36.30 ± 18.65	54.76 ± 7.22	8.95 ± 11.43
Fr-1	81	51.08 ± 1.91	36.62 ± 3.10 *	12.3 ± 3.51	39.68 ± 2.13	53.87 ± 4.74	6.45 ± 2.64
	8.1	49.48 ± 5.20	48.93 ± 3.42	1.56 ± 2.70	47.43 ± 3.48	48.30 ± 2.66 *	4.27 ± 1.75
	1.62	46.99 ± 10.71	38.56 ± 3.45 *	14.45 ± 13.72	52.24 ± 0.56	47.76 ± 0.56 *	0
Fr-2	173	46.88 ± 4.04	51.09 ± 5.90	2.03 ± 2.24	43.51 ± 5.05	55.33 ± 5.69	1.16 ± 2.10
	17.3	41.27 ± 20.96	42.81 ± 23.34	16.02 ± 8.05	37.25 ± 6.56	62.75 ± 6.56	0
	3.46	48.84 ± 2.17	49.19 ± 2.25	1.97 ± 0.36	27.67 ± 4.85	59.59 ± 4.11	12.74 ± 2.55

The third instar silkworms were fed with mulberry leaves that were soaked in the SFV, DSFV, Fr-1 and Fr-2 samples for 7 days, and then fed with mulberry leaves without jellyfish venom to cocoon. Each sample involved three dosages and phosphate buffer solution (0.01 mol/L, pH 6) was employed as control. The experiment involved three replicates per dosage and the control. After 12 and 14 days of treatment, 10 silkworms were randomly taken from each replicate and the contents of chitin, protein, ash and lipid in the cuticle were determined. * *p* < 0.05, ** *p* < 0.01, *n* = 3.

The insect cuticle served a very important role in the growth and development of insects. The integrity of the cuticular structure and function was substantially dependent on the maintenance of chitin in the structure of the cuticle. Chitin was the main component of the insect cuticle, for it represented 20%–50% of the dry weight of the cuticle. Because plants and vertebrates do not contain chitin, chitin can be employed as a safe and strong selective target of insecticides. If chitin was lacking, the layered structure of the cuticle was disturbed, such as the integrity of the procuticle and the assembly of the epicuticle, the detachment of the cuticle from the epithelium, and the influence on the formation of the normal morphology of the epithelium. In addition, the chitin involved in the process of the cuticular pigment and the degree of pigmentation was significantly reduced if the chitin was lacking [28]. A normal distribution of chitin and other processes related to chitin was critical to the synthesis of chitin in the insect cuticle. Any barriers or disorder may lead to the abnormal development of individual insects or embryonic lethality and other very serious consequences [29]. A complex set of biochemical and physiological transformations, such as the hormone regulation of chitin synthetase at the transcriptional and translational levels, posttranslational processing, guidance and transportation at correct sites of chitin synthetase clusters within the cells, the complex process of inserting chitin synthase clusters into the plasma membrane, were included in the synthesis and deposition of chitin [30].

Candy and Kilby firstly proposed a chitin synthesis pathway for insect chitin synthesis that begins with glucose, terminates in uridine diphosphate-*N*-acetylglucosamine (UDP-GlcNAc), and was confirmed in the study of a variety of insects [31]. In general, UDP-GlcNAc was transformed into chitin under the action of chitin synthetase, and the chitin was transported to the epidermis by the transmembrane. Chitin synthetase existed in the form of zymogen; its activity was dependent on the divalent cations Mg^2+^ and Mn^2+^ and required protease to activate [32,33,34]. Jellyfish venom possesses protease activity [35] but the chitin synthesis was inhibited by SFV. We speculated that SFV may inhibite UDP-GlcNAc transmembrane transport, thus blocking the chitin synthetase and UDP-GlcNAc to synthesize the chitin, according to Mitsui demonstrating that Diflubenzuron inhibited the chitin synthesis of cabbage armyworm [36].

### 2.3. Effect of Jellyfish Venom on Silkworm AChE Activity

AChE is a key enzyme in the nerve conduction of insects, and the stimulation of postsynaptic membrane is terminated to ensure the normal transfer of neurotransmitter by degrading acetylcholine [37]. Table 3 shows the effect of jellyfish venom on silkworm AChE activity. Compared with the control, the four samples had no distinct effect on the AChE activity after 12 days of treatment. After 14 days of treatment, the four samples significantly improved the AChE activity. Of the four samples, Fr-2 had the most significant effect (*p* < 0.01), and the AChE activity increased by 3.39, 4.33 and 3.71 times at 173, 17.3, 3.46 µg/mL, respectively. The effect of the samples on the AChE activity had no significant correlation with concentration and was probably related to the physiology of the silkworm. Jellyfish venom is a type of protein with a unique structure [18,20] and its activity may be similar to the activities of AChE, hydrolyzing acetylcholine into acetic acid and choline, which increased the AChE activity. The compounds in jellyfish venom that may bind to the enzyme and the type of activation that is involved required additional studies.

The primary biochemical mode of action of organophosphorus insecticides is the inhibition of AChE activity in animals. The inhibition of AChE in the central nervous system and the consequent blockade of the transfer of nerve impulses across a synapse was the major factor in the death of organophosphorus-poisoned animals. The organophosphorus insecticides had an adverse impact on silkworms, which may cause a drastic reduction in silk production [38]. The use of chemicals for plant protection requires extreme care and a safe period to prevent deleterious effects on non-target species. This study revealed that jellyfish venom can increase AChE activity. If the jellyfish venom is developed as a bioinsecticide, it can be interchangeably employed with organophosphorus insecticides to mitigate the adverse impact on non-target species.

**Table 3:** Effect of jellyfish venom on silkworm acetylcholinesterase (AChE) activity.

Sample	Concentration (µg/mL)	AChE Activity (Μm·min^−1^·mg^−1^)	
		12 days	14 days
Control	0	24.55 ± 6.54	9.65 ± 1.21
SFV	188	16.70 ± 2.70	21.26 ± 9.20
	18.8	26.69 ± 7.12	27.86 ± 18.04
	3.8	18.45 ± 1.96	24.04 ± 5.17 *
DSFV	196	15.98 ± 9.48	13.63 ± 0.71 **
	19.6	21.60 ± 2.78	30.76 ± 13.18
	3.9	12.67 ± 2.97	20.61 ± 1.53 **
Fr-1	81	22.48 ± 7.54	37.72 ± 17.59
	8.1	13.19 ± 1.37	49.66 ± 15.42
	3.6	15.62 ± 3.21	48.70 ± 8.55 *
Fr-2	173	16.15 ± 4.14	32.76 ± 1.58 **
	17.3	19.95 ± 6.59	41.81 ± 4.93 **
	3.5	17.48 ± 8.16	35.87 ± 3.63 **

The third instar silkworms were fed with mulberry leaves that were soaked in the SFV, DSFV, Fr-1 and Fr-2 samples for 7 days, and then fed with mulberry leaves without jellyfish venom to cocoon. Each sample involved three dosages and phosphate buffer solution (0.01 mol/L, pH 6) was employed as control. The experiment involved three replicates per dosage and the control. After 12 and 14 days of treatment, 10 silkworms were randomly taken from each replicate and the AChE were determined. * *p* < 0.05, ** *p* < 0.01, *n* = 3.

## 3. Experimental Section

### 3.1. Animals

A commercial strain of the silkworm, *B. mori* (*Chunlei* × *Zhenzhu*) was obtained from the Qingzhou Shandong (China) Guangtong silkworm Group Co. Ltd.

### 3.2. Venom Preparation

Jellyfish *N. nomurai* specimens were collected in the Aoshan Bay in Qingdao, Shandong Province, China, in August 2012. The bloom of jellyfish has become increasingly more serious and significantly affects tourism, fishing, military affairs and marine sporting events. Thus, fishing for jellyfish is permitted by the department of fisheries in China. Tentacles were manually excised *in vivo*, packed in polythene bags, and immediately frozen at −20 °C. The frozen tentacles were subsequently sonicated in cold (4 °C) phosphate buffer solution (PBS, 0.01 mol/L, pH 6) eight times for 30 s each time at 100 mV. The sample was clarified by centrifugation (15,000 *g*) (Heraeus Fresco 17, Hanau, Germany) for 20 min at 4 °C and employed as full venom (SFV). SFV was repeatedly dialyzed in 0.01 mol/L PBS (pH 6.0) to remove metal ions from jellyfish tentacles and subsquently marked as DSFV. SFV was subjected to 30% (NH_4_)_2_SO_4_ saturation by adding solid (NH_4_)_2_SO_4_ with gentle stirring at 4 °C. The mixture was left for 2 h to enable complete precipitation to occur, and then centrifuged (15,000 *g*) for 20 min at 4 °C. The precipitate (Fr-1) was removed, the supernatant was subjected to 60% (NH_4_)_2_SO_4_ saturation, and the entire process was repeated. Fr-1 and Fr-2 were repeatedly dialyzed in 0.01 mol/L PBS (pH 6.0) to remove (NH4)_2_SO_4_. SFV, DSFV, Fr-1 and Fr-2 were frozen at −20 °C until use. The concentration was determined by the method of Bradford [39], using bovine serum albumin (BSA) as a standard.

### 3.3. Effect of Jellyfish Venom on Silkworm Growth

The third instar silkworms were fed with mulberry leaves that were soaked in the SFV, DSFV, Fr-1 and Fr-2 samples for 7 days, and then fed with mulberry leaves without jellyfish venom to cocoon. Each sample involved three dosages; and PBS (0.01 mol/L, pH 6) was employed as the control. The experiment involved three replicates per dosage and the control. 40 third instar silkworms were utilized in each replicate. The mass of *B. mori* was quantified with an analysis balance, and the mortality was recorded after 2, 4, and 6 days of treatment. The increment rate of mass (IRM) was counted according to the following formula:

IRM% = (mt − m0) × 100/m0
(1)

mt: mass after treatment; m0: mass before treatment.

### 3.4. Effect of Jellyfish Venom on Silkworm Cuticle

The silkworms were treated as previously described. After 12 and 14 days of treatment, 10 silkworms were randomly taken from each replicate. Contents of chitin, protein, ash and lipid in the cuticle were determined according to the following procedures [40]: (1) Silkworms were immersed in boiling water for 1 min to condense the protein. The heads, rumps and feet were sheared, the abdomen was cut along the midline and the muscle tissues and fat in abdomen were erased with absorbent cotton soaked in 70% alcohol; (2) The cuticle was washed with distilled water, placed in a constant weight crucible (m0) and dried to constant weight (m1) at 96–98 °C. The difference between m1 and m0 was the weight of the cuticle; (3) The cuticle was soaked in ether for 10–15 min to remove crude fat, and the ether was changed twice during this process. The cuticle was washed with distilled water and dried to constant weight (m2) at 96–98 °C. The difference between m1 and m2 was the weight of crude fat; (4) The cuticle was soaked in 18% KOH solution for 6 h at 60 °C to remove protein and the 18% KOH solution was changed four times during this process. The cuticle was washed with ethanol and distilled water thrice and dried to a constant weight (m3) at 96–98 °C. The difference between m2 and m3 was the weight of protein; (5) The residual cuticle was incinerated to constant weight (m4) in a muffle furnace for 3 h at 550 °C. The difference between m3 and m4 was the weight of the chitin and the difference between m4 and m0 was the weight of ash.

### 3.5. Effect of Jellyfish Venom on Silkworm AChE Activity

The silkworms were treated as previously described. After 12 and 14 days of treatment, 10 silkworms were randomly taken from each replicate and homogenized in 3 mL of ice-cold phosphate buffer. The resultant fluids were clarified by centrifugation (10,000 *g*) for 20 min at 4 °C and utilized as an enzyme solution (ES). The concentration of ES was determined from the Bradford method [37], using BSA as a standard.

The AChE activity was determined according to the method described by Chen [41]. Eighteen small tubes were divided into six equal groups, and acetylcholine bromide (4 mmol/L) solutions (0.2, 0.4, 0.6, 0.8, 1.0, and 1.0 mL) were added to groups 1–6. Then, the volume of the reaction system calculated to be 1.5 mL by PBS (0.01 mol/L, pH 6). Two milliliters of a mixture of 2 mol/L H_4_ClNO and 3.5 mol/L NaOH solutions (1:1 ratio of temporary mixing) were added to groups 1–5. The reaction systems were shaken for 1 min, and the solutions of 1 mL 4 mol/L HCl were also added to groups 1–5. Then, the reaction systems of groups 1–5 showed different shades of color by adding 1 mL 0.37 mol/L FeCl_3_ solution. The control (the sixth group) was obtained by interchanging the added order of H_4_ClNO and NaOH mixture and HCl. The absorbance was determined at 540 nm. The standard curve was constructed with the volume of acetylcholine bromide in the reaction system and A_540_ as abscissa and ordinate, respectively. The 0.5 mL of ES was added to the reaction system with incubation for 40 min at 37 °C, and the H_4_ClNO and NaOH mixture, HCl and FeCl_3_ were successively added. As a result, the A_540_ value was conveniently determined. The AChE activity was calculated according to the following formula:

*A* (μM·min^−1^·mg^−1^) = *V* × 0.004 × 10^6^ × 2/*C* (2)

*A*: the AChE activity; *V*: the volume of hydrolyzed acetylcholine bromide; *C*: the concentration of ES.

### 3.6. Statistical Analysis

All data were expressed as means ± SD of three parallel measurements. Data were analyzed by software SPSS (IBM, New York, NY, USA) and all tests were considered statistically significant at *p* < 0.05.

## 4. Conclusions

The four samples SFV, DSFV, Fr-1 and Fr-2 influenced the growth, cuticle and AChE activity of *B. mori*. However, no *B. mori* died, and the rest of *B. mori* was able to cocoon. The results concluded that jellyfish venom had no toxic effect on non-target insect silkworm *B. mori* and exhibited acceptable environmental safety. Therefore, as a type of natural substance, jellyfish venom has potential as a biopesticide. Additional experimental investigations are needed to explore the mechanism of the effect of jellyfish venom on chitin and AChE activity.

## References

[1] Li B., Wang Y., Liu H., Xu Y., Wei Z., Chen Y., Shen W. (2010). Resistance comparison of domesticated silkworm (*Bombyx mori* L.) and wild silkworm (*Bombyx mandarina* M.) to phoxim insecticide. Afr. J. Biotechnol..

[2] Peng G., Wang J., Ma L., Wang Y., Cao Y., Shen W., Li B. (2011). Transcriptional characteristics of acetylcholinesterase genes in domestic silkworms (*Bombyx mori*) exposed to phoxim. Pestic. Biochem. Phys..

[3] Gu Z., Zhou Y., Xie Y., Li F., Ma L., Sun S., Wu Y., Wang B., Wang J., Hong F. (2014). The adverse effects of phoxim exposure in the midgut of silkworm, *Bombyx mori*. Chemosphere.

[4] Arakawa T., Yukuhiro F., Noda H. (2011). Subacute and delayed toxicity of iminoctadine liquid formulation, which contains iminoctadine triacetate as an antifungal component on a non-target domesticated insect, the silkworm, *Bombyx mori* L. (Lepidoptera: Bombycidae). Pestic. Biochem. Phys..

[5] Vyjayanthi N., Subramanyam M.V.V. (2002). Effect of fenvalerate-20EC on sericigenous insects I. Food utilization in the late-age larva of the silkworm, *Bombyx mori* L. Ecotoxicol. Environ. Saf..

[6] Phugare S.S., Kalyani D.C., Gaikwad Y.B., Jadhav J.P. (2013). Microbial degradation of imidacloprid and toxicological analysis of its biodegradation metabolites in silkworm (*Bombyx mori*). Biochem. Eng. J..

[7] SEPA (2004). Guidelines for Environmental Safety Assessment Testing of Chemical Pesticides-Silkworm Toxicity Test, National Standard of the People’s Republic of China.

[8] ACIS Guidelines for Preparation of Study Results Submitted When Applying for Registration of Agricultural Chemicals, Section 2-8-2 Silkworm Toxicity Studies.

[9] Omori M., Kitamura M. (2004). Taxonomic review of three Japanese species of edible jellyfish (Scyphozoa: Rhizostomeae). Plankton Biol. Ecol..

[10] Zhang F., Sun S., Jin X., Li C. (2012). Associations of large jellyfish distributions with temperature and salinity in the Yellow Sea and East China Sea. Hydrobiologia.

[11] Shi Y.Q., Sun S., Zhang G.T., Wang S.W., Li C.L. (2014). Distribution pattern of zooplankton functional groups in the Yellow Sea in June: A possible cause for geographical separation of giant jellyfish species. Hydrobiologia.

[12] Yu H., Liu X., Dong X., Xing R., Liu S., Li C., Li P. (2005). Insecticidal activity of proteinous venom from tentacles of jellyfish *Rhopilema esculentum*. Bioorg. Med. Chem. Lett..

[13] Sollod B.L., Wilson D., Zhaxybayeva O., Gogarten J.P., Drinkwater R., King G.F. (2005). Were arachnids the first to use combinatorial peptide libraries?. Peptides.

[14] Morabito R., la Spada G., Crupi R., Esposito E., Marino A. (2015). Crude venom from nematocysts of the jellyfish *Pelagia noctiluca* as a tool to study cell physiology. Cent. Nerv. Syst. Agents Med. Chem..

[15] Corzo G., Diego-Garcia E., Clement H., Peigneur S., Odell G., Tytyat J., Possani L.D., Alagon A. (2008). An insecticidal peptide from the theraposid *Brachypelma smithi* spider venom reveals common molecular features among spider species from different genera. Peptides.

[16] Lipkin A., Kozlov S., Nosyreva E., Blake A., Windass J.D., Grishin E. (2002). Novel insecticidal toxins from the venom of the spider *Segestria florentina*. Toxicon.

[17] Abdel-Rahman M.A., Quintero-Hernandez V., Possani L.D. (2013). Venom proteomic and venomous glands transcriptomic analysis of the Egyptian scorpion *Scorpio maurus palmatus* (Arachnida: Scorpionidae). Toxicon.

[18] Mariottini G.L., Pane L. (2010). Mediterranean jellyfish venoms: A review on scyphomedusae. Mar. Drugs.

[19] Li R., Yu H., Xing R., Liu S., Qing Y., Li K., Li B., Meng X., Cui J., Li P. (2012). Application of nanoLC-MS/MS to the shotgun proteomic analysis of the nematocyst proteins from jellyfish *Stomolophus meleagris*. J. Chromatogr. B.

[20] Li R., Yu H., Xing R., Liu S., Qing Y., Li K., Li B., Meng X., Cui J., Li P. (2012). Isolation, identification and characterization of a novel antioxidant protein from the nematocyst of the jellyfish *Stomolophus meleagris*. Int. J. Biol. Macromol..

[21] Nath B.S. (2003). Shifts in glycogen metabolism in hemolymph and fat body of the silkworm, *Bombyx mori* (Lepidoptera: Bombycidae) in response to organophosphorus insecticides toxicity. Pestic. Biochem. Phys..

[22] Nath B.S. (2000). Changes in carbohydrate metabolism in hemolymph and fat body of the silkworm, *Bombyx mori* L., exposed to organophosphorus insecticides. Pestic. Biochem. Phys..

[23] Nath B.S., Suresh A., Varma B.M., Kumar R.P.S. (1997). Changes in protein metabolism in hemolymph and fat body of the silkworm, *Bombyx mori* (Lepidoptera: Bombycidae) in response to organophosphorus insecticides toxicity. Ecotoxicol. Environ. Saf..

[24] Fan J., Chen X., Hu Q. (2013). Effects of Destruxin A on hemocytes morphology of *Bombyx mori*. J. Integr. Agric..

[25] Li Q., Xu J., Zhang S., Zhang X., Chen A. (2010). Safety evaluation on myricetin and crude extract from Waxberry leaves to non-target organisms. J. Anhui Agric. Sci..

[26] Zhen Z., Yang G., Li Y., Li N., Jia M., Huo Y. (2011). Effect of several medicinal plants on silkworm. North Seric..

[27] Li J., Yan F., He W., Xiao M., Chen Y., Chen F. (2005). Toxicity and mechanism of Jatropherol I to silkworm, *Bombyx mori* L. Chin. J. Pestic. Sci..

[28] Moussian B., Schwarz H., Bartoszewski S., Nüsslein-Volhard C. (2005). Involvement of chitin in exoskeleton morphogenesis in *Drosophila melanogaster*. J. Morphol..

[29] Devine W., Lubarsky B., Shaw K. (2005). Requirement for chitin biosynthesis in epithelial tube morphogenesis. Proc. Natl. Acad. Sci. USA.

[30] Cohen E. (2001). Chitin synthesis and inhibition: A revisit. Pest Manag. Sci..

[31] Candy D.J., Kilby B.A. (1962). Studies on chitin synthesis in desert locust. J. Exp. Biol..

[32] Duran A., Bowers B., Cabib E. (1975). Chitin synthetase zymogen is attached to the yeast plasma membrane. Proc. Natl. Acad. Sci. USA.

[33] Hardy J., Gooday G. (1983). Stability and zymogenic nature of chitin synthase from *Candida albicans*. Curr. Microbiol..

[34] Mayer R., Chen A., Deloach J. (1980). Characterization of a chitin synthase from the stable fly, *Stomoxys*
*calcitrans* (L). Insect Biochem..

[35] Li C., Yu H., Liu S., Xing R., Guo Z., Li P. (2005). Factors affecting the protease activity of venom from jellyfish *Rhopilema esculentum* Kishinouye. Bioorg. Med. Chem. Lett..

[36] Mitsui T., Nobusawa C., Fukami J. (1984). Mode of inhibition of chitin synthesis by diflubenzuron in the cabbage armyworm, *Mamestra brassicae* L. J. Pestic. Sci..

[37] Soreq H., Seidman S. (2001). Acetylcholinesterase-new roles for an old actor. Nat. Rev. Neurosci..

[38] Nath B.S., Kumar R.P.S. (1999). Toxic impact of organophosphorus insecticides on acetylcholinesterase activity in the silkworm, *Bombyx mori* L. Ecotoxicol. Environ. Saf..

[39] Bradford M.A. (1976). Rapid and sensitive method for the quantitation of microgram quantities of protein utilizing the principle of protein-dye binding. Anal. Biochem..

[40] Chi D., Miao J., Han J., Shen Z., Shao P. (1997). Study on the mechanism of triflumuron on the *Hyphantria cunea*. For. Sci. Tech..

[41] Chen C. (1996). Insect Physiological and Biochemical Experiments.

